# Wu-Mei-Wan enhances brown adipose tissue function and white adipose browning in obese mice via upregulation of HSF1

**DOI:** 10.1186/s13020-024-01053-2

**Published:** 2025-01-03

**Authors:** Shen Chen, Kexin Nie, Hongzhan Wang, Yang Gao, Xinyue Jiang, Hao Su, Zhi Wang, Yueheng Tang, Fuer Lu, Hui Dong, Jingbin Li

**Affiliations:** 1 Institute of Integrated Traditional Chinese and Western Medicine, Tongji Hospital, Tongji Medical College, Huazhong University of Science and Technology, Wuhan, 430030 Hubei China; 2 Department of Integrated Traditional Chinese and Western Medicine, Tongji Hospital, Tongji Medical College, Huazhong University of Science and Technology, Wuhan, 430030 Hubei China

**Keywords:** Wu-Mei-Wan, Obesity, Heat shock factor 1, White adipose tissue, Brown adipose tissue

## Abstract

**Background:**

This research aims to explore the anti-obesity potential of Wu-Mei-Wan (WMW), particularly its effects on adipose tissue regulation in obese mice induced by a high-fat diet (HFD). The study focuses on understanding the role of heat shock factor 1 (HSF1) in mediating these effects.

**Methods:**

HFD-induced obese mice were treated with WMW. Body weight, food intake, and histopathological analysis of adipose tissue were conducted. Brown adipose tissue (BAT) activity was evaluated using Positron Emission Tomography, and ultrastructural changes were examined via transmission electron microscopy. Proteomic analysis identified targets of WMW in obesity treatment. HSF1 expression was inhibited to confirm its role. Molecular docking studied interactions between WMW and HSF1. Short-chain fatty acids (SCFAs) in the intestines were measured to determine if WMW’s effects on HSF1 are mediated through SCFAs. Protein expression was assessed using western blot, immunohistochemistry, immunofluorescence and RT-qPCR were employed to detect the mRNA levels. Statistical analyses included t-tests, ANOVA, and non-parametric tests like the Mann–Whitney U test or Kruskal–Wallis test.

**Results:**

WMW significantly mitigates the adverse effects of a HFD on body weight and glucose metabolism in obese mice. Both low-dose WMW and high-dose WMW treatments led to reduced weight gain and improved glucose tolerance, with low-dose WMW showing more pronounced effects. WMW also reversed structural damage in BAT, enhancing mitochondrial integrity and thermogenic function, particularly at the low dose. Additionally, WMW treatment promoted the browning of WAT, evidenced by increased expression of key thermogenic proteins such as UCP1 and PGC-1α. The increase in HSF1 expression in both BAT and WAT, observed with WMW treatment, was crucial for these beneficial effects, as inhibition of HSF1 negated the positive outcomes. Furthermore, WMW treatment led to elevated levels of short-chain fatty acids SCFAs in the intestines, which are associated with increased HSF1 expression.

**Conclusions:**

WMW represents a potent therapeutic strategy for obesity, promoting metabolic health and beneficial modulation of adipose tissue through an HSF1-dependent pathway.

**Supplementary Information:**

The online version contains supplementary material available at 10.1186/s13020-024-01053-2.

## Introduction

Obesity is a complex metabolic disorder primarily characterized by excessive adipose tissue accumulation, arising from a sustained imbalance between energy intake and expenditure [1]. Beyond mere weight augmentation, it entails a myriad of metabolic pathway disruptions, chronic inflammation, and an elevated risk profile for cardiovascular and metabolic diseases [2]. The therapeutic landscape encompasses lifestyle modifications, pharmacotherapeutic interventions, and surgical options. Lifestyle modifications, comprising dietary regulation and regular physical activity, form the cornerstone of obesity management due to their foundational and low-risk nature, albeit requiring persistent adherence and manifesting gradual effectiveness [3]. Pharmacotherapy aims at appetite suppression or reduction in fat absorption, facilitating more rapid weight reduction but associated with potential side effects, including gastrointestinal discomfort, cardiovascular complications, metabolic dysregulation, psychological impacts, urinary infections, and risks of dependency and weight rebound upon prolonged usage [4]. Bariatric procedures, such as gastric bypass and sleeve gastrectomy, offer substantial weight loss but bear significant risks, costs, and potential for malnutrition, surgical complications, gastroesophageal reflux, alongside implications for psychological and long-term digestive health [5]. Thus, identifying efficacious and safer obesity treatments remains a pressing priority.

White adipose tissue (WAT), the predominant fat form in obesity, serves chiefly as an energy store, accumulating lipids predominantly as triglycerides [6]. Excess energy prompts white adipocytes to enlarge via augmented lipid storage, occasionally surpassing their healthy expansion threshold, culminating in cellular dysfunction and adipose tissue inflammation [7]. At the molecular level, WAT modulates whole-body energy balance and metabolic homeostasis by secreting a spectrum of hormones and cytokines, including adiponectin, leptin, and inflammatory mediators [8]. Obesity disrupts the equilibrium of these secretions, potentially leading to systemic metabolic disorders, notably insulin resistance and type 2 diabetes [9]. Moreover, the role of WAT extends beyond mere energy storage, influencing appetite regulation, energy dissipation, and immune and inflammatory responses, thereby rendering its dysfunction central to obesity and related metabolic pathologies [10]. On the other hand, brown adipose tissue (BAT), a specialized adipose depot, has attracted significant attention for its unique role in energy metabolism and weight management. Contrasting with WAT's energy storage function, BAT exhibits heightened metabolic activity, rapidly oxidizing stored fats to generate heat, a process crucial for thermoregulation. BAT cells are structurally distinct, enriched with mitochondria harboring uncoupling protein 1 (UCP1), a pivotal element in thermogenesis [11]. UCP1 disrupts the mitochondrial electron transport chain from ATP synthesis, channeling chemical energy directly into heat production [12]. Such an energy dissipation mechanism underscores BAT’s vital role in thermoregulation and energy equilibrium. In obese individuals, diminished BAT quantity and activity contribute to reduced energy expenditure, furthering energy surplus and weight gain [13].

The therapeutic horizon in obesity management contemplates the activation of BAT or enhancement of white fat browning to augment its metabolic activity, representing a promising avenue for intervention. BAT’s functionality is influenced by an array of physiological and pathological stimuli, including ambient temperature, dietary intake, and hormonal signals from thyroid and adrenal glands, intimately connected to energy expenditure and weight regulation [14, 15]. Interventions, pharmacological or through lifestyle modification like heightened physical activity or cold exposure, aim to boost BAT’s quantity and function, thus increasing systemic energy dissipation and offering novel insights into obesity management [15]. However, the distribution and activity of BAT in adults, particularly among the obese and elderly, are generally limited, with impaired metabolic capacity under obese conditions, leading to attenuated thermogenic response and accentuated energy storage and weight accretion [16]. Effectively harnessing or augmenting BAT’s quantity and functionality to bolster its role in energy consumption and weight regulation remains an area of intense research focus. Emerging studies have illuminated that specific pharmacological and nutritional interventions can enhance BAT functionality, laying the groundwork for environmental and biological strategies to harness BAT's potential in obesity management [17].

In addition to inherent BAT, white adipocytes can undergo browning or beiging, a sophisticated, tightly regulated physiological process converting them into metabolically active brown-like fat cells. This metamorphosis involves an array of molecular and cellular mechanisms, encompassing transcription factors, signaling pathways, and hormonal modulation. Beiged white adipocytes exhibit increased mitochondrial quantity and activity, swiftly utilizing energy to generate heat [18]. Compared to energy-storing white fat, this transition offers a novel avenue for enhancing bodily energy dissipation. Research has underscored that white fat browning may confer multiple metabolic benefits, including improved insulin sensitivity, glucose handling, lipid profile amelioration, and possibly retarding atherosclerosis progression [19]. Consequently, white fat browning holds promise not only in obesity management but also as a therapeutic target for related metabolic disorders, including type 2 diabetes, hypertension, and cardiovascular diseases. Yet, effectively inducing white fat browning remains an elusive and crucial scientific endeavor. Various external stimuli, including cold exposure, physical exercise, and certain drugs and nutrients, have been recognized to induce white fat browning to varying degrees, with differential effectiveness and feasibility among individuals [20, 21]. Moreover, although some pharmaceuticals and bioactive molecules have demonstrated potential in promoting white fat browning, they frequently accompany adverse reactions or uncertain long-term outcomes [22].

In traditional Chinese medicine (TCM), obesity is frequently linked to the accumulation of phlegm-dampness. The excessive intake of rich, fatty, and sweet foods impairs the small intestine's ability to distinguish between the pure and impure, while also disrupting the large intestine’s function of transmission. This dysfunction leads to the buildup of phlegm-dampness, which, in turn, weakens spleen yang, resulting in a cold and deficient state in both the spleen and stomach [23, 24]. It may also stagnate and transform into heat. Thus, obesity in TCM is characterized by a mix of cold and heat within the digestive system [25]. The treatment approach combines strategies to address both cold and heat conditions. Wu-Mei-Wan (WMW), a classical Chinese herbal formula, epitomizes this dual approach in treating digestive disorders.

First described in the “Treatise on Febrile Diseases Caused by Cold” (Shang Han Za Bing Lun) around AD 200–210, WMW comprises ten medicinal herbs including *Prunus mume* (Sieb.) Sieb. & Zucc. (Wumei), *Asarum heterotropoides f. mandshuricum* (Maxim.) Kitag. (Xixin), *Zingiber officinale* Roscoe (Ganjiang), *Coptis chinensis* Franch. (Huanglian), *Angelica sinensis* (Oliv.) Diels (Danggui), *Typhonium giganteum* Engl. (Fuzi), *Zanthoxylum bungeanum* Maxim. (Shujiao), *Cinnamomum cassia* (L.) J. Presl (Guizhi), *Panax ginseng* C. A. Mey. (Renshen), and *Phellodendron amurense* C. K. Schneid. (Huangbai). According to the “Authentic Transmission of Medical Principles” by Zheng Shouquan during the Qing Dynasty, WMW is a representative formula in Traditional Chinese Medicine for treating disorders of mixed cold and heat (also known as Jueyin disease), which is suitable for the pathogenesis of obesity [26, 27]. Recent clinical studies have demonstrated the effectiveness of WMW in weight reduction [28]. Our preliminary research suggests that WMW may combat obesity by inhibiting the formation of white adipose tissue while promoting the development and activity of brown adipose tissue [25, 29]. Additionally, our prior studies have shown that WMW can alleviate obesity in mice by modulating gut microbiota [30]. Moreover, modern pharmacological research has identified that key components of WMW, such as cinnamaldehyde, ginsenoside Rb1, citric acid, and berberine, possess weight-reducing properties and can improve insulin resistance [31–34]. However, the specific targets of WMW on adipose tissue require further investigation. In this context, we are particularly focused on ascertaining whether WMW can augment the activity and quantity of brown fat and induce the browning of white fat, thereby contributing to obesity management.

## Methods

### Herbal preparation

The preparation of WMW was consistent with our previous studies [35]. All herbs, obtained from Beijing Tcmages Pharmaceutical Co., Ltd., were used in the preparation of the WMW decoction, conducted weekly in our laboratory. Initially, the herbs (net weight: 96 g) were soaked in 500 ml of water for 1 h. Following this, the herb mixture underwent a 2-h boiling process, with Typhonii Rhizoma preboiled separately for the same duration. Subsequently, the concentrated herb mixture (1.92 g/ml, approximately 50 ml) was obtained using a rotary evaporator. High-dose WMW (25 ml) was then diluted to 0.96 g/ml (approximately 25 ml) using sterile water. Thus, 25 ml of high-dose WMW and 25 ml of low-dose WMW were prepared weekly.

### Animal experimental design and sample collection

This study was conducted in accordance with the Guidelines for Regulation for the Administration of Affairs concerning Experimental Animals. Specific pathogen-free (SPF) 6-week-old male C57BL/6 mice were procured from Shulaibao Biotechnology Corporation and housed within the animal barrier system at Tongji Medical College of Huazhong University of Science and Technology, Wuhan, China. Institutional Animal Care and Use Committee approval was obtained from Tongji Hospital, Tongji Medical College, Huazhong University of Science and Technology, Wuhan, China, prior to conducting the experiments (No. 2915). Following a 1-week acclimation period, mice were assigned to either a normal chow diet (ND) or a high-fat diet (HFD, 60% calories from fat; Research Diet, Mediscience Ltd, China) for a duration of 16 weeks. The study employed a two-phase experimental design to comprehensively assess the effects of WMW on HFD-induced obesity. The C57BL/6 mice were categorized as follows:

#### Phase one

ND + Vehicle (standard diet with daily gavage of 10 ml/kg 0.9% saline solution), HFD + Vehicle (HFD with daily gavage of 10 ml/kg 0.9% saline solution), HFD + LWMW (HFD with daily gavage of 0.96 g/ml WMW at a volume of 10 ml/kg), HFD + HWMW (HFD with daily gavage of 1.92 g/ml WMW at a volume of 10 ml/kg) (6 mice in each group).

#### Phase two

ND + Vehicle (standard diet with daily gavage of 10 ml/kg 0.9% saline solution and intraperitoneal injection of 10 ml/kg 0.9% saline solution), HFD + Vehicle (HFD with daily gavage of 10 ml/kg 0.9% saline solution and intraperitoneal injection of 10 ml/kg 0.9% saline solution), HFD + LWMW (HFD with daily gavage of 0.96 g/ml WMW at a volume of 10 ml/kg and intraperitoneal injection of 10 ml/kg 0.9% saline solution), HFD + LWMW + Inhibitor (HFD with daily gavage of 0.96 g/ml WMW at a volume of 10 ml/kg and intraperitoneal injection of DTHIB (25 mg/kg, Catalog No. S9806, Selleck) at a volume of 10 ml/kg) (6 mice in each group).

Throughout the experiment, mice were housed in a controlled environment (22–24 °C, 50–60% humidity, 12-h light cycles) with ad libitum access to food and water. Regular records were maintained for body weight and food intake. Food intake was measured using the food consumption method). At the beginning of each feeding period, the initial weight of the food provided was recorded, and at the end of the designated time interval (e.g., 24 or 48 h), the remaining food was weighed. The difference between the initial and remaining food weight was calculated as the food intake. To account for any potential spillage or food wastage, the leftover food scattered outside the food hopper was also collected and weighed. The final consumption values were adjusted accordingly. In group housing, food intake per mouse was estimated by dividing the total food consumption by the number of mice in the cage. At the conclusion of the experiment, mice were euthanized, and samples of colon contents, WAT, BAT, and liver were swiftly collected. Some samples were fixed in 4% paraformaldehyde or 2.5% glutaraldehyde solution, while others were snap-frozen and stored at −80 °C for subsequent analysis.

### Glucose tolerance test (OGTT) and insulin sensitivity test (ITT)

#### OGTT

After a 12-h fast, mice received an oral administration of a glucose solution (0.75 g/kg body weight). Blood glucose levels were measured at 15, 30, 60, 90, and 120 min post-administration.

#### ITT

Following a 4-h fast, mice were intraperitoneally injected with insulin (0.75 U/kg body weight). Blood glucose levels were measured at similar time intervals post-injection.

Blood glucose measurements were conducted using a blood glucose monitor and glucose strips (Johnson & Johnson).

### RT-qPCR

Total RNA was extracted from mouse tissues using an RNA extraction kit, followed by cDNA synthesis using reverse transcriptase and random primers. Specific primers were used to amplify corresponding genes, with SYBR Green used for quantification of the amplified products to analyze changes in gene expression levels. The primer sequences for the amplification of various genes were meticulously designed and are as follows: UCP1, with a 5′ → 3′ sense of GCTTTGCCTCACTCAGGATTGG and a 3′ → 5′ sense of CCAATGAACACTGCCACACCTC; PGC1α, featuring a 5′ → 3′ sense of TATGGAGTGACATAGAGTGTGCT and a 3′ → 5′ sense of CCACTTCAATCCACCCAGAAAG; PPARα, with a 5′ → 3′ sense of AGAGCCCCATCTGTCCTCTC and a 3′ → 5′ sense of ACTGGTAGTCTGCAAAACCAAA; SIRT1, having a 5′ → 3′ sense of GCTGACGACTTCGACGACG and a 3′ → 5′ sense of TCGGTCAACAGGAGGTTGTCT; ELOVL3, with a 5′ → 3′ sense of TTCTCACGCGGGTTAAAAATGG and a 3′ → 5′ sense of GAGCAACAGATAGACGACCAC; CIDEA, featuring a 5′ → 3′ sense of TGACATTCATGGGATTGCAGAC and a 3′ → 5′ sense of GGCCAGTTGTGATGACTAAGAC; CIDEC, with a 5′ → 3′ sense of ATGGACTACGCCATGAAGTCT and a 3′ → 5′ sense of CGGTGCTAACACGACAGGG; TBX1, having a 5′ → 3′ sense of CTGTGGGACGAGTTCAATCAG and a 3′ → 5′ sense of TTGTCATCTACGGGCACAAAG; PRDM16, with a 5′ → 3′ sense of CCAAGGCAAGGGCGAAGAA and a 3′ → 5′ sense of AGTCTGGTGGGATTGGAATGT; HSF1, featuring a 5′ → 3′ sense of AACGTCCCGGCCTTCCTAA and a 3′ → 5′ sense of AGATGAGCGCGTCTGTGTC.

### Histopathological analysis

Tissue samples were immediately fixed in 4% paraformaldehyde solution for 24 h, followed by dehydration in a graded ethanol series and clearing in xylene. The cleared tissues were embedded in paraffin, sectioned into 4–5 µm slices, and stained with Hematoxylin and Eosin. The slides were then mounted with a neutral resin and examined under a microscope to observe histopathological morphology.

### Mouse PET scanning

Mice were fasted for at least 6 h (allowed free access to water) to reduce background noise and improve image quality. Mice were injected with the radioactive tracer FDG (Fluorodeoxyglucose) via tail vein and then kept in a quiet environment for 45–60 min before scanning. Proper anesthesia was administered to ensure immobility during the scan. Mice were then placed in a PET scanner for whole-body imaging. PET images were quantitatively analyzed to assess tracer uptake in the interscapular region, indicating the activity of BAT.

### Transmission electron microscopy (TEM) of BAT

BAT samples were immediately fixed in 2.5% glutaraldehyde solution at 4 °C overnight. Fixed samples were dehydrated in a graded ethanol series, infiltrated with low concentration resin followed by high concentration resin for embedding. Ultrathin sections were cut, stained with lead, placed on grids, and observed under a transmission electron microscope. Lipid droplet size, number, and mitochondrial count were quantified using ImageJ, and mitochondrial ultrastructural changes were assessed.

### Data dependent acquisitioned (DDA) and data independent acquisition (DIA) mass spectrometry analysis

In the methods section for protein extraction, approximately 100 mg of frozen samples were rapidly ground into a fine, uniform powder using liquid nitrogen. These samples were then homogenized in 1 ml of phenol extraction buffer, followed by the addition of 1 ml saturated phenol with Tris–HCl (pH 7.5). After vigorous shaking and a 30-min incubation at 4 °C, the mixture was centrifuged at 7100*g* for 10 min at 4 °C to separate the upper phenolic phase from the aqueous phase. The phenolic phase was then transferred to a new tube and mixed with five volumes of pre-cold 0.1 M ammonium acetate in methanol and stored at − 20 °C overnight. Post-centrifugation at 12,000*g* for 10 min at 4 °C, the precipitated protein pellet was washed twice with pre-cold methanol and twice with ice-cold acetone, then air-dried and resuspended in 300 μl lysate solution. The solution was incubated for 3 h at room temperature, centrifuged to remove insoluble material, and the supernatant containing total extractable protein was collected. Protein concentrations were determined using the bicinchoninic acid assay.

For SDS-PAGE, 10 μg of protein was subjected to 12% SDS-PAGE separation, followed by staining with Kaumas Brilliant Blue using eStain LG (GenScript, Nanjing). Gel analysis was conducted using an automatic digital gel image analysis system (Tanon, Shanghai).

Protein digestion was standardized by diluting proteins from each sample to the same concentration and volume, with 50 μg of protein treated with 25 mM DTT to a final concentration of approximately 5 mM and incubated at 55 °C for 30–60 min. This was followed by cooling and addition of iodoacetamide to a final concentration of about 10 mM, and incubated in the dark at room temperature for 15–30 min. Proteins were precipitated with pre-cooled acetone and stored at − 20 °C overnight. After centrifugation at 8000*g* for 10 min at 4 °C, the acetone was evaporated, and the protein pellet was redissolved in 100 μl NH_4_HCO_3_ solution (50 mM). An appropriate volume of enzymatic digestion buffer was added (protein:enzyme ratio of 50:1), and digestion was carried out at 37 °C for 12 h or overnight, stopped by adjusting pH to 3 with phosphoric acid. Desalting was performed using SOLA™ SPE before drying under vacuum, resuspension, and addition of iRT peptides.

High-pH reversed-phase fractionation involved separating pooled sample peptides using an 1100 HPLC System (Agilent) with a specific gradient of mobile phases A (2% acetonitrile in water) and B (98% acetonitrile in water). The peptides were collected at set intervals and lyophilized for mass spectrometry analysis.

Mass spectrometry was performed using DDA and DIA on a TimsTOF Pro2 mass spectrometer (Bruker), with peptide separation on a C18 column using an EASY-nLCTM 1200 system (Thermo, USA) and specific linear gradients. The DDA MS data were acquired using the PASEF method with specified parameters, while DIA employed 56 windows with tailored collision energy. Database searches for protein identification were conducted with Spectronaut Pulsar™ using default settings, and DIA data were analyzed against a constructed spectral library.

Statistical analyses identified differentially expressed proteins (DEPs), with thresholds for fold change and P-value set to discern significance. Protein annotations were performed using GO and KEGG databases, and protein–protein interaction analysis utilized the String database.

### Spectral library construction and data analysis

DDA data were analyzed with Maxquant (v 1.6.6.0) and searched against the Swiss Prot database (Mouse; 17022) with cysteine alkylation as a static modification and methionine oxidation and N-terminal acetylation as variable modifications. FDR filtering was employed using a target-decoy strategy. DIA data were processed with Skyline, imported into Spectronaut along with the spectral library, and analyzed using non-linear iRT calibration and dynamic window prediction. Transition parent and child ion charges were set, and the 6 strongest ions were extracted for peptide quantification. The mProphet algorithm was employed for FDR filtering, and relative protein quantitation was obtained using the MSstats R package. The quality of peptide identification was ensured by the distribution of peptide lengths meeting specific quality control criteria.

### Immunohistochemistry

The paraffin-embedded sections were deparaffinized in xylene and rehydrated through a graded alcohol series. Antigen retrieval was performed using citrate buffer (pH 6.0) under high temperature and pressure. Endogenous peroxidase activity was quenched with hydrogen peroxide. Non-specific binding was blocked using a suitable blocking serum. The sections were then incubated with primary antibodies specific to the target proteins at 4 °C overnight. After washing, the sections were incubated with biotinylated secondary antibodies followed by the addition of an avidin–biotin–peroxidase complex. Color development was achieved using a chromogen such as DAB. Counterstaining was performed with hematoxylin, and the sections were dehydrated, cleared, and mounted. The slides were examined under a light microscope, and the expression of the target proteins was assessed qualitatively and quantitatively.

### Immunofluorescence detection

Following the immunohistochemistry, paraffin-embedded tissue sections were processed through deparaffinization, hydration, and antigen retrieval. The sections were then blocked to prevent non-specific binding and incubated with primary antibodies overnight at 4 °C. After primary incubation, the sections were washed and incubated with fluorophore-conjugated secondary antibodies for 1–2 h at room temperature. Nuclei were counterstained with DAPI. The slides were finally mounted using an anti-fade mounting medium and examined under a fluorescence microscope. The fluorescence intensity was quantified using ImageJ software to assess the expression levels of the target proteins.

### Western blot

In brief, protein extraction, electrophoresis and transmembrane were implemented, which were strictly in accordance with the standard procedure of western blot. Then, a nitrocellulose (NC) membrane was immersed in solution of primary antibodies including HSF1 (Abcam), HNRNPA2B1 (ABclonal), PGC1α (ABclonal), UCP1 (ABclonal), HSP40 (ABclonal), HSP70 (ABclonal), HSP90 (Abcam) for the whole night at the temperature of 4 °C. Next, NC membrane was immersed in solution of secondary antibody including IRDye 800cw goat anti-rabbit IgG (lot no. CST, Danvers, MA, USA) and goat anti-mouse IgG (ab216772, abcam). Finally, the NC membrane was scanned by Odyssey (LI-COR Biosciences, Lincoln, NE, USA).and protein band intensity was quantified using ImageJ, normalized against the expression of the internal control protein β-actin (ABclonal).

### SCFAs analysis

In this study, all chemicals and solvents were of analytical or HPLC grade, including water, methanol, formic acid, and acetonitrile, which were sourced from Thermo Fisher Scientific. Analytical reagent-grade 3-nitrophenylhydrazine (3NPH) ·HCl (97%), N-(3-dimethylaminopropyl)-Nʹ-ethylcarbodiimide (EDC)·HCl, and standards were purchased from Sigma-Aldrich. Sample preparation involved creating standard solutions and calibration curves, with single standard stock solutions prepared at 1 mg/ml in water and mixed to form primary mixed standard stock solutions (MSS), which were then diluted to appropriate concentrations for calibration. Tissue or fecal samples underwent pre-treatment and metabolite extraction tailored to the chemical characteristics of the targets, involving grinding with steel balls, ultrasonic extraction, and centrifugation. Derivatization of standards and samples was achieved by reacting with 3NPH and EDC in acetonitrile, followed by UPLC-MS/MS analysis using a Nexera UHPLC LC-30A and an AB SCIEX SelexION Triple Quad™ 5500 System, with conditions optimized for each analyte in multiple reaction monitoring (MRM) mode.

### Molecular docking analysis

The 3D structure of HSF1 was retrieved from the Protein Data Bank (PDB, ID: 2ldu) and the structures of cinnamaldehyde (ID: 637511), ginsenoside Rb1 (ID: 3086007), citric acid (ID: 311), berberine (ID: 2353), hesperidin (ID: 10621), coptisine (ID: 72322), jatrorrhizine (ID: 72323), aconitine (ID: 245005), 6-gingerol (ID: 442793), and phellodendrine (ID: 3081405) were downloaded from PubChem. Before performing molecular docking, the receptor protein was optimized by adding hydrogens, removing existing ligands, processing disulfide bonds, and eliminating water molecules. Molecular docking was conducted using MOE and PyMOL software, focusing on the conformations with the lowest binding energies for each compound.

### Data analysis

Differences between two groups were compared using the t-test, while ANOVA was employed to assess significant differences among groups with more than two categories. If the data did not satisfy the assumptions of normal distribution or homogeneity of variances, non-parametric statistical methods such as the Mann–Whitney U test (for two independent samples) or Kruskal–Wallis test (for multi-group comparisons) were used to ensure the validity and applicability of the statistical analyses.

## Results

### Effects of WMW on body weight and glucose-lipid metabolism in obese mice

**Fig. 1:**
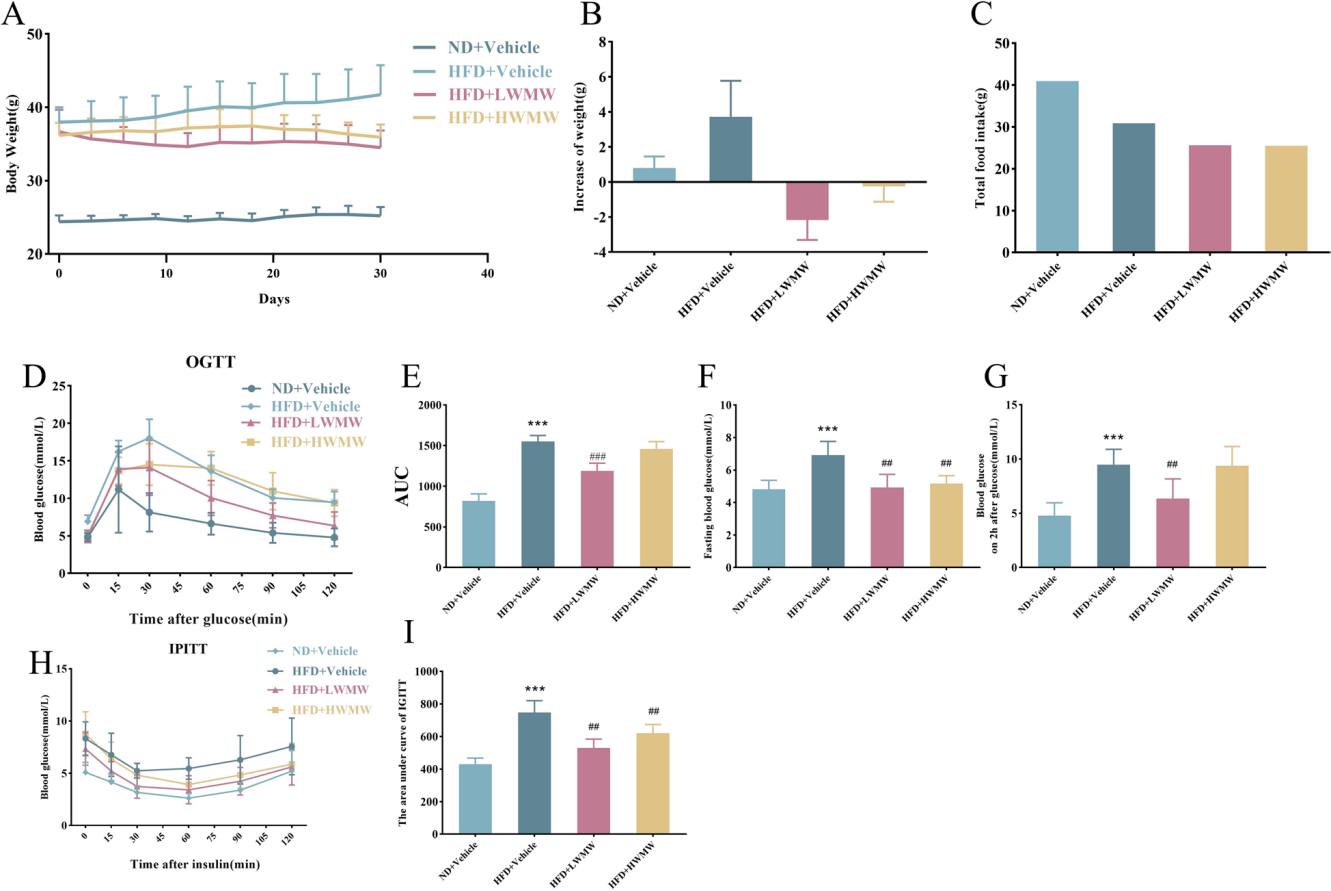
WMW reduces body weight, improves glucose tolerance, and modifies intestinal metabolites in obese mice. **A** Body weight monitoring of mice every 3 days over the treatment period. **B** Change in body weight compared to initial weight. **C** Food intake measurement throughout the experiment. **D** Oral Glucose Tolerance Test (GTT) in mice after overnight fasting with administration of 0.75 g/kg glucose. **E** Area Under the Curve (AUC) for GTT indicating glucose tolerance. **F** Fasting blood glucose levels after an overnight fast. **G** Blood glucose levels 2 h after administration of 0.75 g/kg glucose. **H** Intraperitoneal Insulin Tolerance Test (ITT) conducted after 6 h of fasting with administration of 1 U/kg insulin. **I** Area Under the Curve (AUC) for ITT indicating insulin sensitivity. (Statistical significance indicated as compared to ND + Vehicle group: p < 0.05 (*), p < 0.01 (**); and compared to HFD + Vehicle group: p < 0.05 (#), p < 0.01 (##); (n = 5)

To determine whether WMW administration could prevent weight gain induced by HFD in obese mice, we utilized two different dosages of WMW treatment over a 4-week period. The body weight and food intake were recorded throughout the treatment duration. These WMW dosages have been proven non-toxic in our prior experiments. Results indicated that compared to the normal diet control group (ND + Vehicle), mice on the HFD (HFD + Vehicle group) showed a significant increase in weight gain rate, confirming the substantial promotion of weight gain by a high-fat diet (Fig. 1A). Conversely, mice treated with both low-dose Wu-Mei-Wan (LWMW) and high-dose Wu-Mei-Wan (HWMW) in the context of a high-fat diet exhibited a reduced average weight gain when compared to the HFD + Vehicle group (Fig. 1B). Food intake showed a slight reduction in mice treated with LWMW and HWMW during this process (Fig. 1C). Overall, while the high-fat diet significantly promoted weight gain, the application of WMW effectively mitigated this process.

Furthermore, WMW treatment also demonstrated positive effects on glucose and lipid metabolism. We assessed glucose metabolism through Oral Glucose Tolerance Tests (OGTT) and Intraperitoneal Insulin Tolerance Tests (IPITT). Blood glucose concentrations were recorded at 0, 15, 30, 60, 90, and 120 min following oral glucose or intraperitoneal insulin administration, and curves were plotted (Fig. 1D and H). The area under the curve (AUC) (Fig. 1E and I), fasting blood glucose levels (Fig. 1F), and 2-h post-oral glucose solution values (Fig. 1G) were statistically analyzed. Results showed that the high-fat diet increased the AUC, fasting blood glucose levels, and 2-h post-oral glucose solution values. LWMW treatment significantly reduced the AUC (Fig. 1E). Further analysis indicated that both concentrations of WMW reduced fasting blood glucose levels (Fig. 1F), but only LWMW reduced the 2-h post-oral glucose solution values (Fig. 1G), indicating a more pronounced effect of LWMW on improving glucose metabolism. IPITT results showed that, after intraperitoneal insulin injection, blood glucose levels in the HFD + Vehicle group were significantly higher than those in the ND + Vehicle group. Similarly, both low-dose and high-dose WMW treatment significantly lowered blood glucose levels, suggesting that WMW enhances insulin sensitivity. In summary, comprehensive analysis indicates that WMW has potential in regulating body weight and glucose-lipid metabolism. The results support the role of WMW in mitigating the adverse effects of a high-fat diet on metabolic health.

### WMW reverses the structural and functional damage in BAT caused by a high-fat diet

**Fig. 2:**
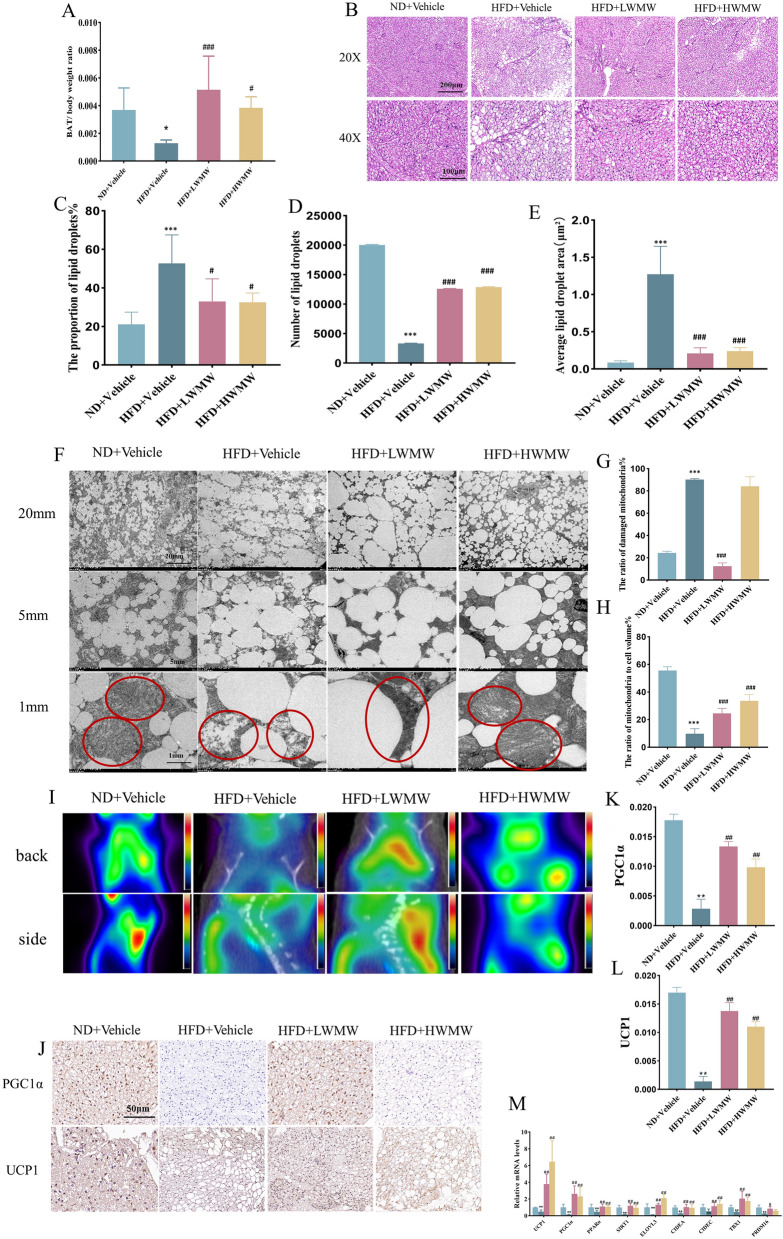
WMW increases the proportion of BAT and preserves its structure and function in obese mice. **A** BAT weight ratio indicating its proportion in body weight. **B**–**E** Representative H&E staining of mice BAT, scale bar, 200 μm and 100 μm (**B**) with Image Pro Plus quantification of lipid droplet area (**C**), number of lipid droplets per field (**D**), and average area per lipid droplet (**E**). **F**–**H** Transmission electron microscopy (TEM) images of BAT, scale bar, 20 mm, 5 mm and 1 mm (**F**); quantification of damaged mitochondria count (**G**) and proportion of damaged mitochondria (**H**). **I** PET imaging of mice BAT. **J**–**L** Immunohistochemical staining for PGC1α and UCP1, scale bar, 50 μm (**J**), with Image Pro Plus quantification of average fluorescence intensity for PGC1α (K) and UCP1 (**L**). **M** RT-qPCR detection of thermogenesis-related gene expression in BAT. Statistical significance is indicated as compared to the ND + Vehicle group: p < 0.05 (*), p < 0.01 (**); and compared to HFD + Vehicle group: p < 0.05 (#), p < 0.01 (##); (n = 5)

BAT is a critical component in thermogenesis, energy expenditure, and the regulation of body weight and obesity [36]. The quantity or proportion of brown fat in the body significantly influences energy consumption. Individuals with a higher amount of brown fat exhibit higher basal metabolic rates, indicating more calories burned at rest, which can prevent weight gain [37]. Given that the most well-known and largest depot of brown fat in mice is located between the shoulder blades, known as the interscapular brown fat pad, we harvested brown fat from this region for weighing and subsequent experiments [38]. We also calculated the BAT/Weight ratio. Results showed that compared to ND + Vehicle, the BAT/Weight ratio significantly decreased in HFD + Vehicle. In contrast, this ratio increased in the HFD + LWMW and HFD + HWMW groups, with a more pronounced difference in the HFD + LWMW group, indicating that WMW mitigates obesity by increasing the proportion of brown fat in the body (Fig. 2A).

Structurally, brown fat differs from WAT. It contains a high number of mitochondria, and the integrity and density of these mitochondria determine its thermogenic capacity and thus its effectiveness in combating obesity [39]. To measure the impact of WMW on brown fat structure, we employed H&E staining to detect lipid droplets in brown fat and conducted a statistical analysis of their area, number, and average size (Fig. 2B–D). TEM was used to observe mitochondria, quantifying the proportion of damaged mitochondria and their area coverage (Fig. 2E–H). Results showed that a high-fat diet led to structural damage in brown fat, characterized by increased lipid droplet accumulation, lipid droplet fusion, mitochondrial structural damage, and reduced numbers. Low-dose WMW treatment effectively reversed these phenomena, while high-dose WMW showed significant improvements in all aspects except for mitochondrial damage.

The primary function of brown fat is non-shivering thermogenesis, a process of burning calories to produce heat, particularly evident in cold environments [40]. To assess the impact of WMW on the function of brown fat, we exposed mice to a 4 °C environment for 4 h followed by PET imaging analysis. Results indicated that compared to the ND + Vehicle group, the interscapular BAT of the HFD + Vehicle group markedly decreased, while both HFD + LWMW and HFD + HWMW groups exhibited higher metabolic activity, with the HFD + LWMW group showing significantly higher activity than the HFD + HWMW group (Fig. 2I). Immunohistochemistry was employed to detect the expression of thermogenesis-related proteins, and results were consistent with PET imaging analysis (Fig. 2J–L). Further, we assessed the mRNA expression levels of several key metabolic regulatory proteins in BAT. Compared to the ND + Vehicle group, the majority of protein mRNA levels significantly declined in the HFD + Vehicle group. Mice treated with WMW showed a marked restoration or enhancement in most protein mRNA levels (Fig. 2M).

In summary, WMW increases the thermogenic function of BAT in obese mice by enhancing the transcription and expression of thermogenesis-related factors.

### WMW increases the browning of WAT in obese mice

WAT is the primary form of fat in the body, comprising the majority of fat stores [41]. The “browning” process of WAT refers to the transformation of white fat cells into a state resembling the characteristics of brown fat cells, which can increase energy expenditure, improve metabolic health, and reduce fat mass [42]. Uncoupling protein 1 (UCP1) is a hallmark protein of brown fat responsible for thermogenesis instead of ATP generation. The browning of WAT is typically accompanied by an increase in UCP1 expression [43]. Peroxisome proliferator-activated receptor gamma coactivator 1-alpha (PGC-1α) is a critical regulator of energy metabolism and mitochondrial biogenesis, and its activation is associated with the browning of adipose tissue [44].

**Fig. 3:**
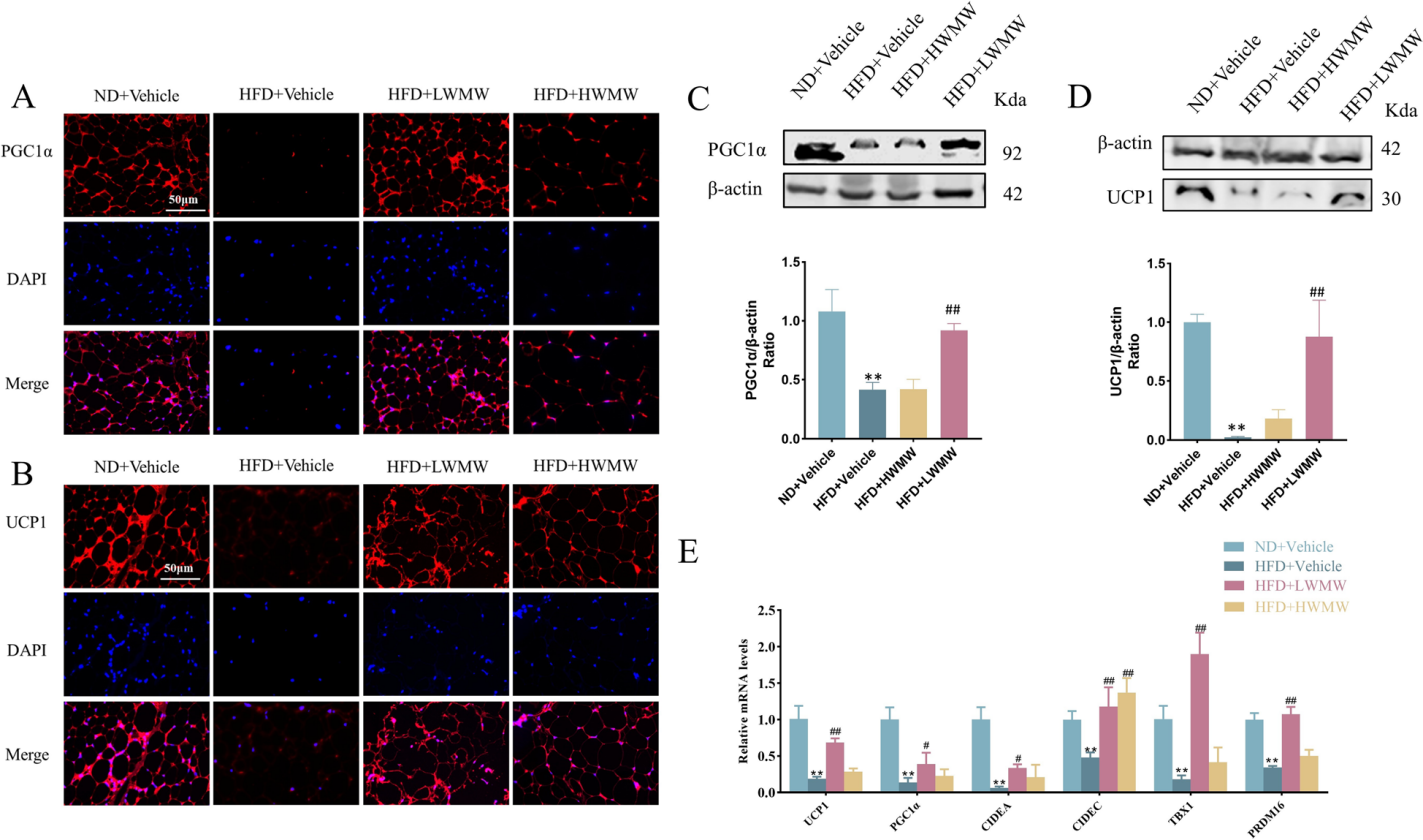
WMW increases the browning of WAT in obese mice. **A**, **B** Immunofluorescence detection of PGC1α (**A**) and UCP1 (**B**) in WAT. **C** Western Blot analysis and quantification of PGC1α in WAT. **D** Western Blot analysis and quantification of UCP1 in WAT. **E** RT-qPCR detection of browning marker genes in WAT. Statistical significance is indicated as compared to the ND + Vehicle group: p < 0.05 (*), p < 0.01 (**); and compared to HFD + Vehicle group: p < 0.05 (#), p < 0.01 (##); (n = 5)

To measure the browning of WAT, we harvested WAT from the epididymal region of obese mice and conducted subsequent analyses. Immunofluorescence and Western blot were employed to detect the expression of UCP1 and PGC-1α proteins, while quantitative PCR was used to measure the transcription of UCP1, PGC-1α, and other genes related to energy metabolism, fatty acid oxidation, and mitochondrial function. Results indicated that a high-fat diet significantly increased the epididymal fat weight ratio and suppressed the browning of WAT in mice. Although WMW did not significantly reduce the epididymal fat weight ratio in mice fed a high-fat diet, low-dose WMW markedly increased the gene transcription and protein expression of UCP1 and PGC-1α (Fig. 3A–D). Additionally, it significantly enhanced the transcription of genes related to energy metabolism, fatty acid oxidation, and mitochondrial function in WAT (Fig. 3E). In contrast, high-dose WMW did not exhibit significant therapeutic potential.

In summary, low-dose WMW treatment effectively promotes the browning of WAT in obese mice. This browning is evidenced by increased expression of key thermogenic and mitochondrial proteins, indicating enhanced energy expenditure and metabolic activity in white fat cells. The findings suggest that WMW, particularly at low doses, may offer a valuable strategy for inducing beneficial changes in WAT, contributing to obesity management and improved metabolic health.

### WMW increases the expression of HSF1 in both white and BAT of obese mice

**Fig. 4:**
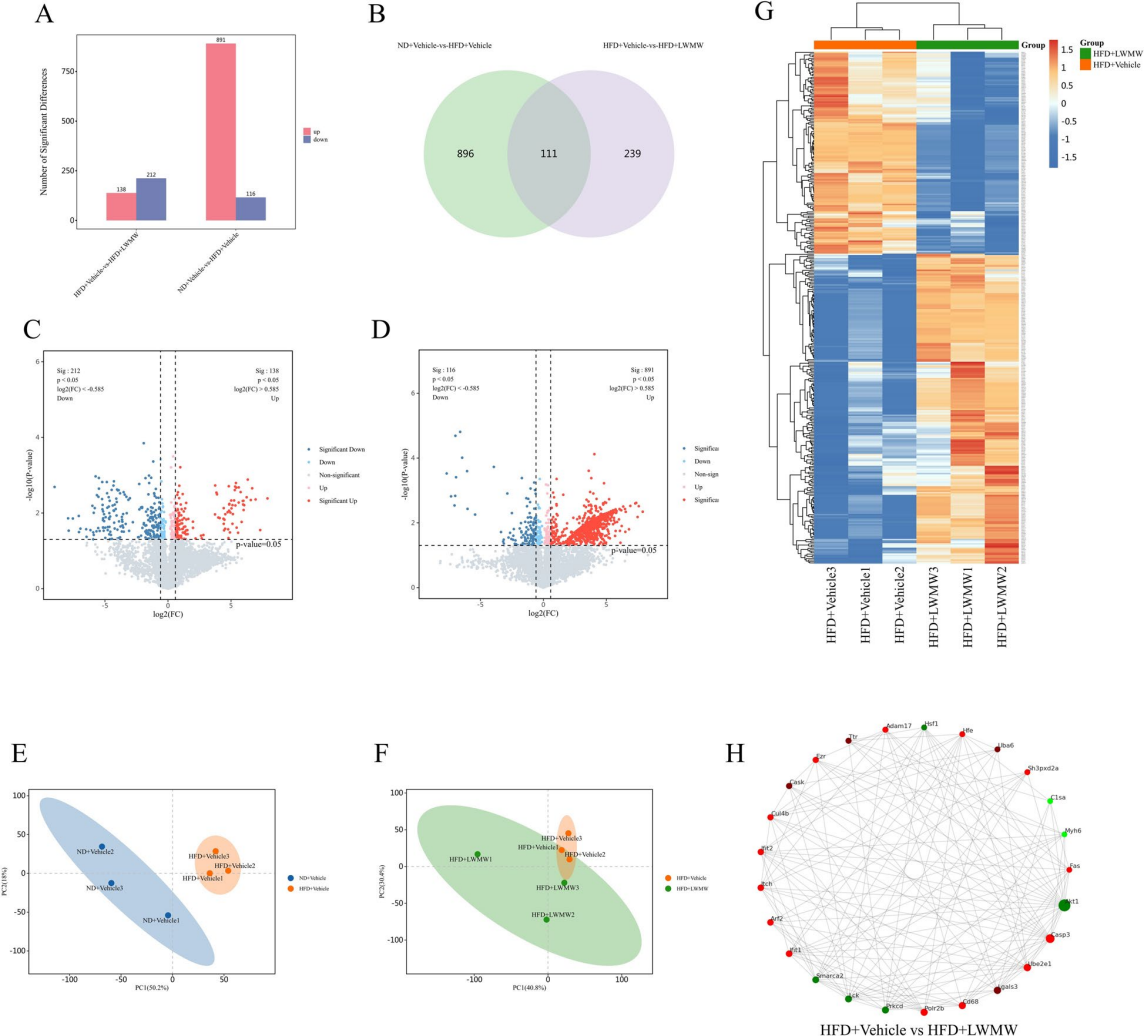
Identification and Quantitative Analysis of Differentially Expressed Proteins (DEPs). **A** Total number of DEPs in two-group comparison. **B** Venn diagram of DEPs and their overlaps. **C**, **D** Volcano plots exhibiting the quantitative protein expression in HFD + Vehicle mice from ND + Vehicle (**C**) and HFD + Vehicle + LWMW (**D**). **E**, **F** PCA plots showing ideal discrimination in HFD + Vehicle mice from ND + Vehicle (**E**) and HFD + Vehicle + LWMW (**F**). **G** Cluster heatmap of expression patterns in HFD + Vehicle and HFD + Vehicle + LWMW groups. **H** Interaction network graph of the top 25 proteins ranked by connectivity among the DEPs between HFD + Vehicle and HFD + Vehicle + LWMW groups

As previously mentioned, low-dose WMW showed better therapeutic potential; hence, we selected the HFD + Vehicle + LWMW group for subsequent experiments to elucidate the mechanisms through which WMW ameliorates obesity in mice by affecting BAT. We conducted LC–MS/MS proteomic analysis on ND + Vehicle, HFD + Vehicle, and HFD + Vehicle + LWMW groups, identifying 58,776 peptides and 6079 proteins. Principal component analysis (PCA) clearly distinguished all nine samples into three main groups, indicating ideal reproducibility within each group (Fig. 4E, F).

When comparing ND + Vehicle mice with HFD + Vehicle mice, 1,007 differentially expressed proteins (DEPs) were quantified (891 upregulated and 116 downregulated) (Fig. 4C), while 350 DEPs (138 upregulated and 212 downregulated) were quantified when comparing HFD + Vehicle with HFD + Vehicle + LWMW mice (Fig. 4D). These proteins were filtered with a P < 0.05 and fold change > 1.5 criteria, with at least two biological replicates (Fig. 4A). Among these, 111 DEPs overlapped between the two comparisons (Fig. 4B).

We analyzed the proteins differentially expressed between ND + Vehicle and HFD + Vehicle mice, obtaining interaction networks for the top 25 connected proteins (Fig. 4G, H). Since our previous experiments suggested that WMW’s therapeutic effects on obesity are likely related to BAT metabolism and thermogenesis, we focused on identifying targets involved in thermogenesis. Among the top 25 connected proteins, we did not find any proteins that were directly involved in the KEGG thermogenesis pathway. After further literature review, we discovered that HSF1 is directly associated with brown adipose tissue thermogenesis [45]. Therefore, we prioritized HSF1 for further study.

**Fig. 5:**
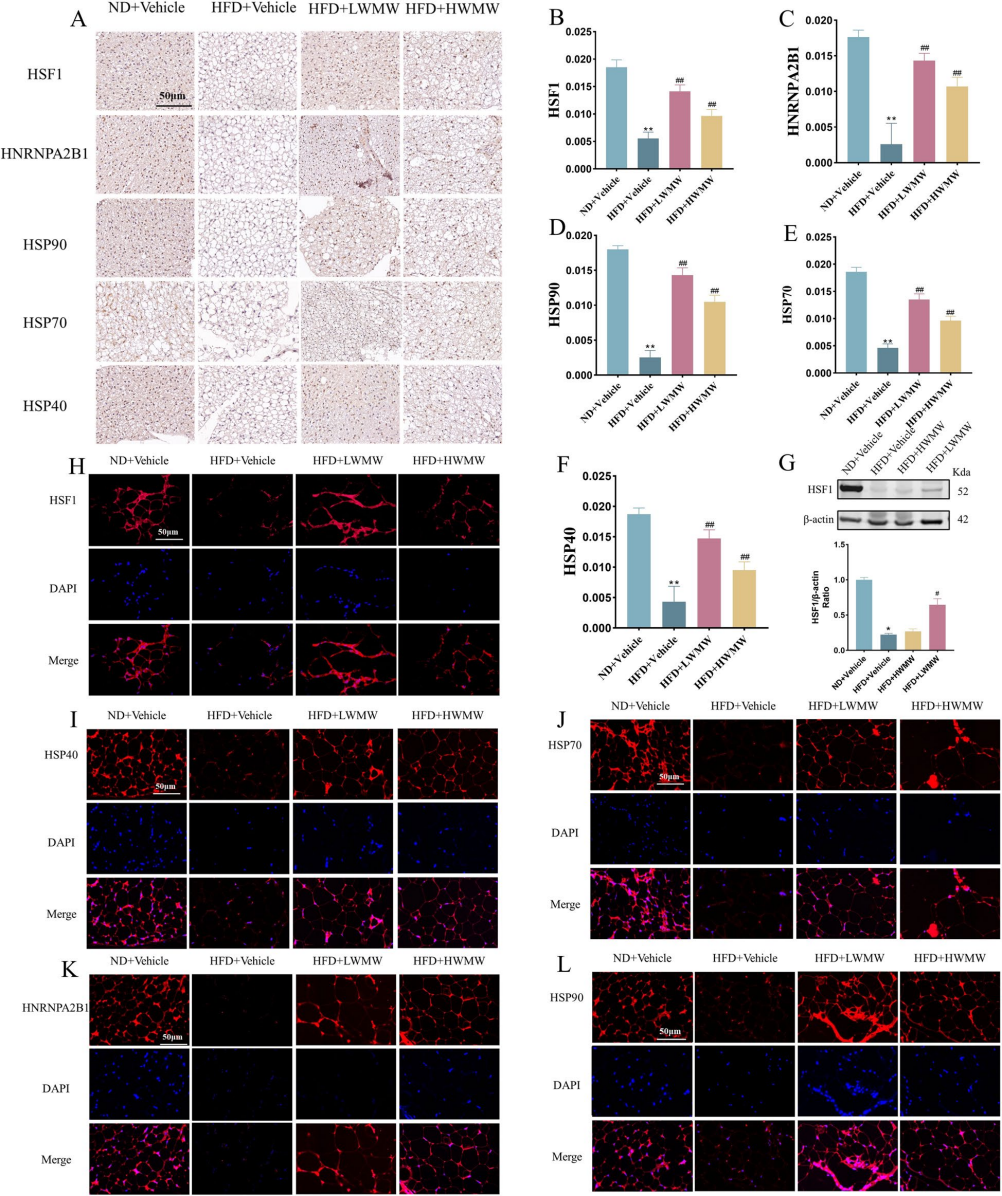
WMW increases expression of HSF1 and related signaling pathway proteins in obese mice. **A**–**F** Immunohistochemical detection of HSF1, HNRNPA2B1, HSP90, HSP70, and HSP40 in BAT with scale bar, 50 μm (**A**); quantitative analysis of HSF1 (**B**), HNRNPA2B1 (**C**), HSP90 (**D**), HSP70 (**E**), and HSP40 (**F**). **G** Western Blot analysis and quantitative assessment of HSF1 in WAT. **H**–**L** Immunofluorescence detection of HSF1 (**H**), HNRNPA2B1 (**I**), HSP90 (**J**), HSP70 (**K**), and HSP40 (**L**) in WAT with scale bar, 50 μm. Statistical significance is indicated as compared to the ND + Vehicle group: p < 0.05 (*), p < 0.01 (**); and compared to HFD + Vehicle group: p < 0.05 (#), p < 0.01 (##); (n = 5)

Existing literature confirms that HSF1 is activated through dissociation and polymerization with Heat Shock Proteins (HSPs) such as HSP90, HSP70, and HSP40, as well as through protein modifications, subsequently activating the thermogenic program in BAT [46]. HSF1 also regulates the transcription of Hnrnpa2b1 (A2B1), enhancing the stability of mRNA for key metabolic genes in brown fat [45]. We used immunohistochemistry to validate the effects of WMW on the expression of HSF1, A2B1, HSP90, HSP70, and HSP40 in BAT. Results indicated that high-fat feeding significantly suppressed the expression levels of these proteins in mice, while both LWMW and HWMW treatments notably increased their expression (Fig. 5A–F). Notably, the increase was more pronounced with LWMW, but HWMW also showed significant upregulation. These data suggest that the role of WMW in increasing the proportion of BAT and preserving its structural and functional integrity may be related to the upregulation of HSF1 expression.

To explore whether WMW also promotes the browning of WAT through increased HSF1 expression, we employed immunofluorescence and Western blot to detect the expression levels of HSF1 and related signaling proteins in WAT. The trend of changes observed was consistent with that in BAT, indicating that WMW’s promotion of white fat browning might be associated with the enhancement of HSF1 expression (Fig. 5G–L). However, while LWMW significantly increased the expression of HSF1 in WAT, HWMW did not show a significant effect on HSF1 expression in WAT. Both doses, however, effectively increased the expression of other related signaling proteins in WAT. This comprehensive approach demonstrates that WMW induces an increase in HSF1 expression, integral to the browning process and the potential therapeutic modulation of adipose tissue in obesity.

### Inhibitor of HSF1 blocks the regulatory effects of WMW on WAT and BAT

**Fig. 6:**
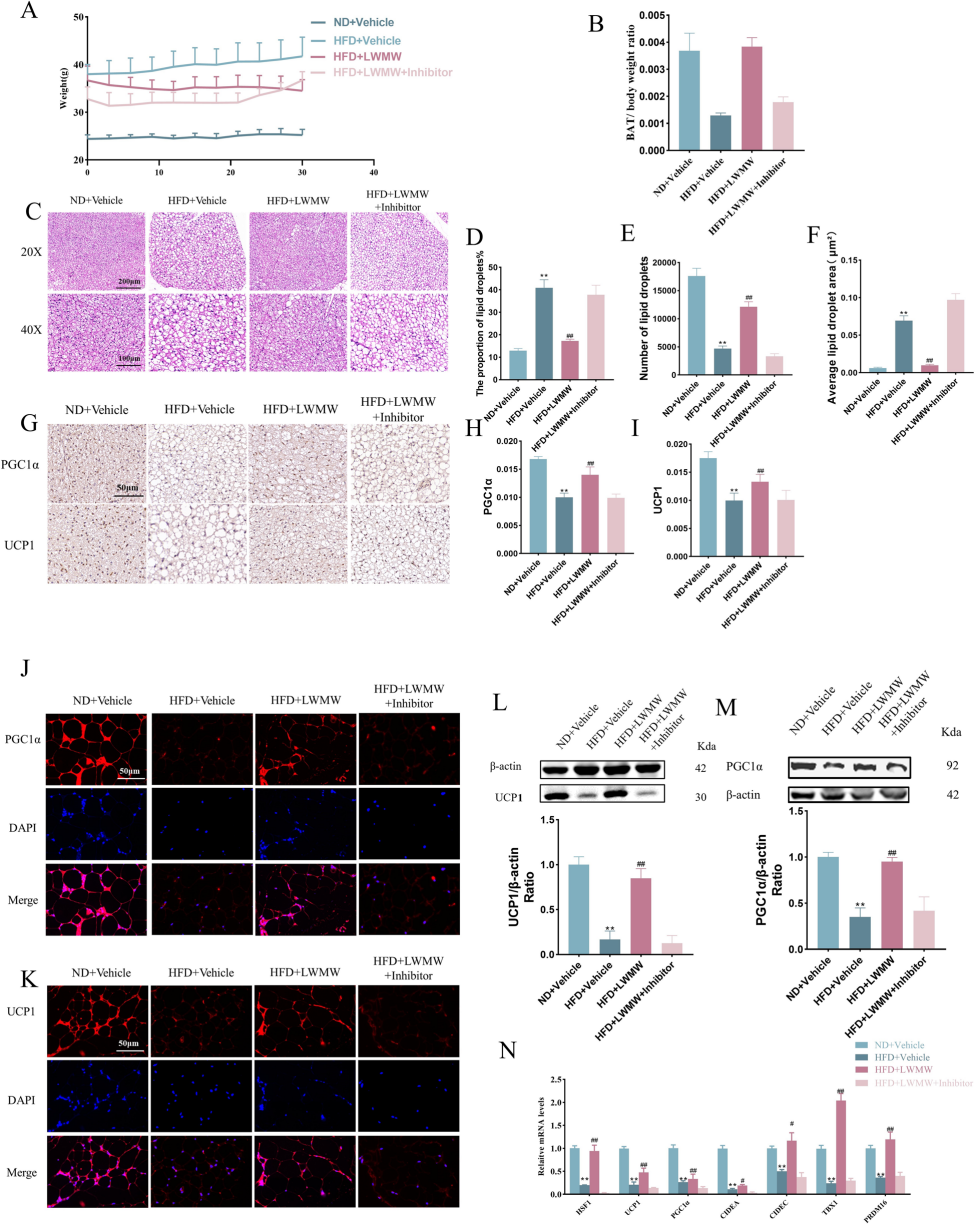
Inhibitor of HSF1 blocks the regulatory effects of WMW on WAT and BAT. **A** Body weight monitoring of mice every 3 days over the treatment period. **B** BAT weight ratio indicating its proportion in body weight. **C**–**F** Representative H&E staining of mice BAT, scale bar, 200 μm and 100 μm (**C**) with Image Pro Plus quantification of lipid droplet area (**D**), number of lipid droplets per field (**E**), and average area per lipid droplet (**F**). **G**–**I** Immunohistochemical staining for PGC1α and UCP1, scale bar, 50 μm (**G**), with Image Pro Plus quantification of average fluorescence intensity for PGC1α (**H**) and UCP1 (**I**). **J**, **K** Immunofluorescence detection of PGC1α (**J**) and UCP1 (**K**) in WAT. **L** Western Blot analysis and quantification of PGC1α in WAT. **M** Western Blot analysis and quantification of UCP1 in WAT. **N** RT-qPCR detection of thermogenesis-related gene expression in BAT. Statistical significance is indicated as compared to the ND + Vehicle group: p < 0.05 (*), p < 0.01 (**); and compared to HFD + Vehicle group: p < 0.05 (#), p < 0.01 (##); (n = 5)

**Fig. 7:**
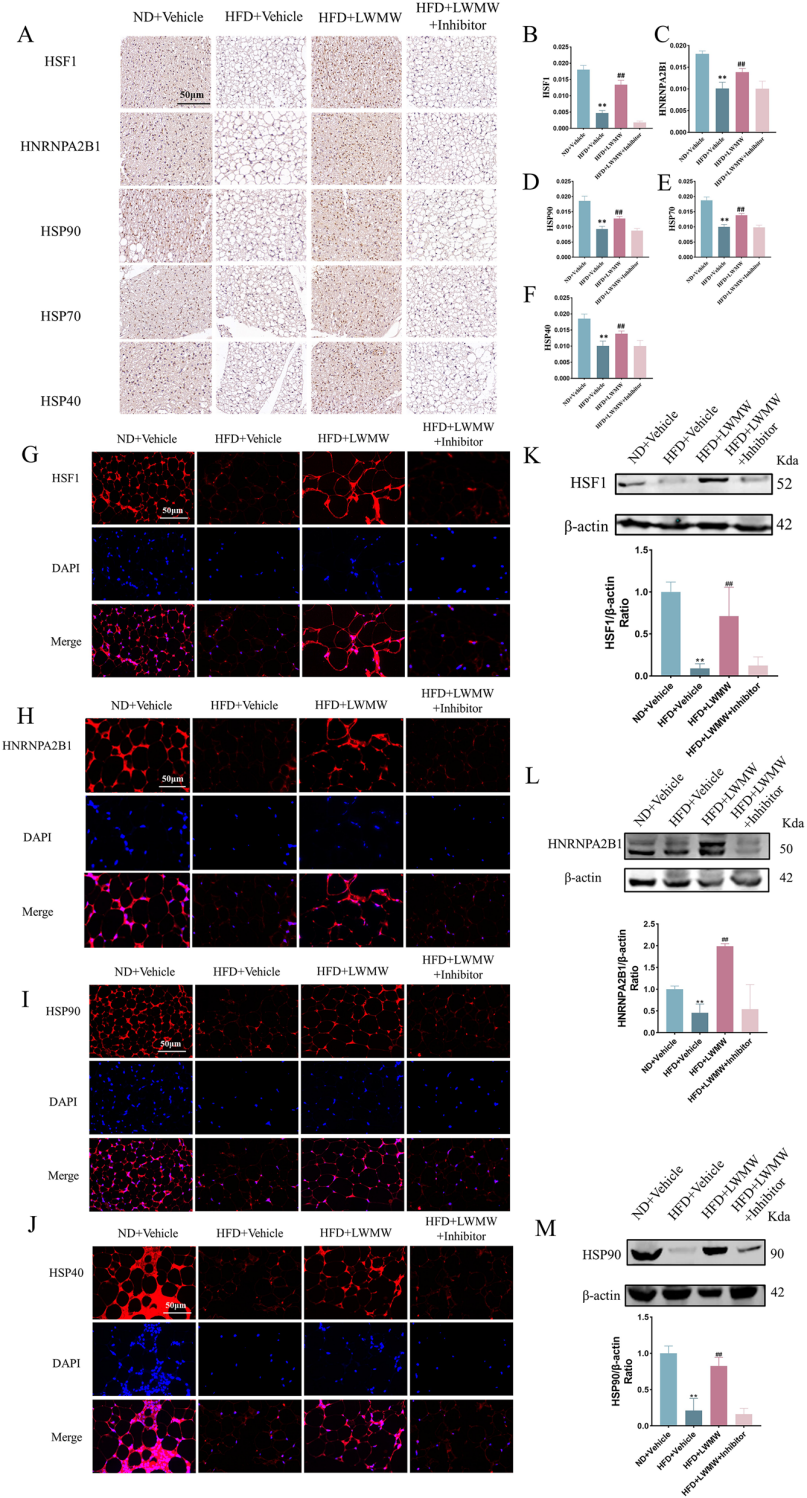
Inhibitor of HSF1 blocks the regulatory effects of WMW on WAT and BAT. **A**–**F** Immunohistochemical detection of HSF1, HNRNPA2B1, HSP90, HSP70, and HSP40 in BAT with scale bar, 50 μm (**A**); quantitative analysis of HSF1 (**B**), HNRNPA2B1 (**C**), HSP90 (**D**), HSP70 (**E**), and HSP40 (**F**). **G**–**J** Immunofluorescence detection of HSF1 (**G**), HNRNPA2B1 (**H**), HSP90 (**I**), HSP40 (**J**) in WAT with scale bar, 50 μm. **K**–**M** Western Blot analysis and quantitative assessment of HSF1 (**K**), HNRNPA2B1 (**L**) and HSP90 (**M**) in WAT. Statistical significance is indicated as compared to the ND + Vehicle group: p < 0.05 (*), p < 0.01 (**); and compared to HFD + Vehicle group: p < 0.05 (#), p < 0.01 (##); (n = 5)

To further ascertain that HSF1 is a target for WMW in the treatment of obesity, and in conjunction with previous studies, we chose DTHIB as an inhibitor of HSF1 [47]. Animal study results showed that the obesity treatment effects of LWMW were diminished in the presence of the inhibitor. Detailed histological staining and protein expression analyses, including HE staining, immunohistochemistry, immunofluorescence, and Western blot, revealed that the LWMW-induced increase in brown fat proportion and the protective effects on brown fat structure and function were blocked by the addition of the HSF1 inhibitor (Fig. 6A–N). Immunohistochemistry, immunofluorescence, and Western blot analyses demonstrated that DTHIB effectively inhibited the action of LWMW in elevating the expression of HSF1 and its pathway-related proteins in both BAT and WAT of obese mice (Fig. 7A–M).

Immunohistochemistry analysis (Fig. 7A) revealed that the expression of HSF1, HNRNPA2B1, HSP90, HSP70, and HSP40 were significantly elevated in the HFD + LWMW group compared to the HFD + Vehicle group. However, these expressions were markedly reduced in the presence of the HSF1 inhibitor (HFD + LWMW + Inhibitor group), suggesting that DTHIB successfully inhibits the effect of LWMW on these proteins. Quantitative analysis of immunohistochemistry results (Fig. 7B–F) further confirmed these findings, showing statistically significant differences in protein expression levels between the groups. Immunofluorescence analysis (Fig. 7G–J) supported these observations, where the fluorescence intensity of HSF1, HNRNPA2B1, HSP90, and HSP40 were higher in the HFD + LWMW group compared to the HFD + Vehicle group, but significantly reduced when the HSF1 inhibitor was present. These results were corroborated by the Western blot analysis (Fig. 7K–M), which showed elevated protein levels of HSF1, HNRNPA2B1, and HSP90 in the HFD + LWMW group that were diminished by the HSF1 inhibitor. This further substantiates that HSF1 is a critical target through which WMW exerts its therapeutic effects on obesity, indicating the importance of the HSF1 pathway in the browning process and overall metabolic regulation.

### WMW enhances SCFA levels in obese mice

**Fig. 8:**
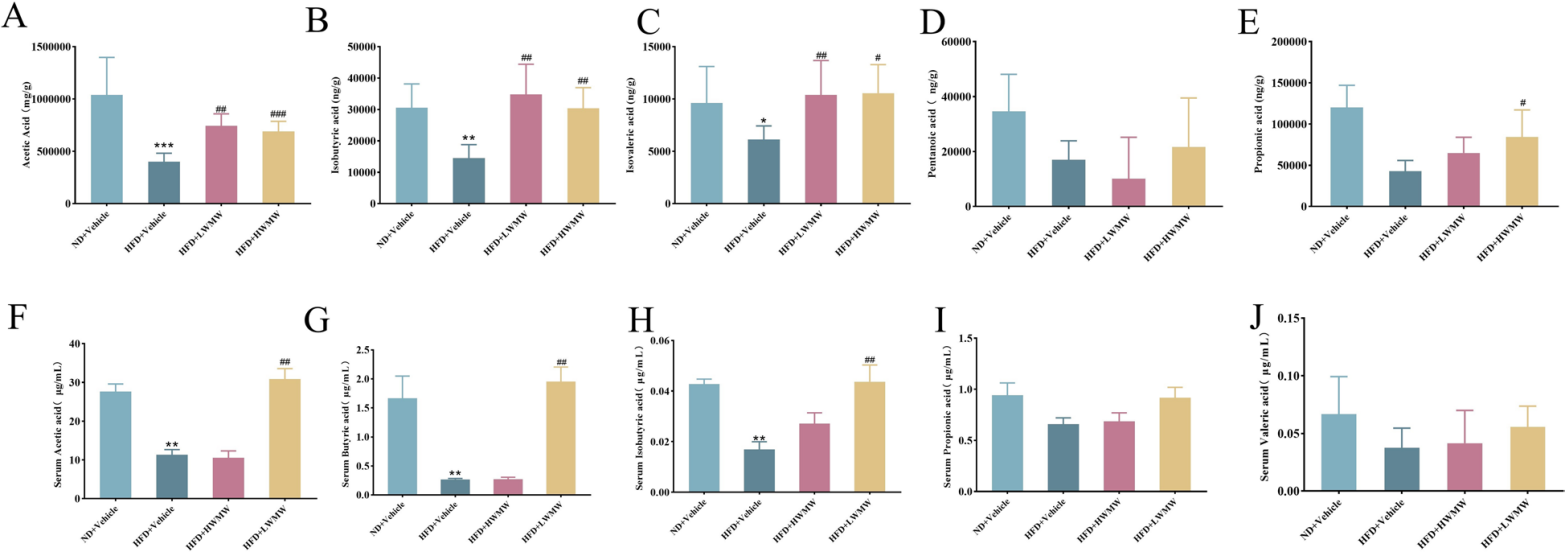
Concentration of short-chain fatty acids (SCFAs) in the colonic contents (**A**–**E**) and serum (**F**–**J**) of mice. The SCFAs measured include acetic acid (**A**, **F**), isobutyric acid (**B**, **G**), isovaleric acid (**C**, **H**), pentanoic acid (**D**, **I**), and propionic acid (**E**, **J**). Statistical significance is indicated as compared to the ND + Vehicle group: p < 0.05 (*), p < 0.01 (**); and compared to HFD + Vehicle group: p < 0.05 (#), p < 0.01 (##); (n = 5)

Previous studies have found that short-chain fatty acids (SCFAs), particularly butyrate, can increase the expression of HSF1 [48]. To determine whether the regulatory effect of WMW on HSF1 is mediated through SCFAs, we analyzed the SCFAs in both the intestinal contents and serum of mice treated with WMW. The results showed that, compared to the normal control group, the levels of acetate, butyrate, and isobutyrate in the intestines of obese mice were significantly reduced, with no significant differences observed for other SCFAs (Fig. 8A–E). Similarly, LWMW significantly increased the levels of these SCFAs in both the intestines and serum of obese mice, showing consistent trends across tissues (Fig. 8F–J). These findings indicate that WMW can enhance SCFA levels in obese mice, potentially contributing to its regulatory effect on HSF1.

### High binding affinity of WMW constituents with HSF1

In our further exploration of the potential constituents within WMW that might interact with HSF1, our preliminary investigations have delineated a compendium of eleven chemical entities present within WMW, including cinnamaldehyde, ginsenoside Rb1, citric acid, berberine, hesperidin, coptisine, jatrorrhizine, aconitine, 6-gingerol, and phellodendrine [35]. Notably, cinnamaldehyde and ginsenoside Rb1 were identified as the predominant constituents, thereby warranting their prioritization in subsequent molecular docking analyses with HSF1.

The outcomes of these docking studies revealed a pronounced binding affinity of both cinnamaldehyde (ΔG: − 4.9 kcal/mol) and ginsenoside Rb1 (ΔG: − 6.2 kcal/mol) towards HSF1, suggesting their significant potential in modulating HSF1 activity (Fig. S1A, B). Extending our investigations to the remaining nine chemical components, molecular docking results consistently demonstrated strong binding affinities with HSF1 across this broader chemical spectrum. Specifically, citric acid exhibited a binding affinity of ΔG: − 4.5 kcal/mol, berberine ΔG: − 6.7 kcal/mol, hesperidin ΔG: − 6.9 kcal/mol, coptisine ΔG: − 7.3 kcal/mol, jatrorrhizine ΔG: − 6.2 kcal/mol, aconitine ΔG: − 5.5 kcal/mol, 6-gingerol ΔG: − 4.6 kcal/mol, and phellodendrine ΔG: − 6.3 kcal/mol (Fig. S1C–J). These findings suggest that WMW may regulate HSF1 activity through direct binding of its various constituents to HSF1.

## Discussion

Obesity has become a global health crisis. Predictions indicate that by 2030, over one billion people worldwide will be affected by it [49]. WMW has shown great potential for clinical application in obesity treatment due to its minimal side effects and higher safety profile.

Classical theory suggests that “excess weight leads to obstruction of the stomach, and excessive sweetness impairs the spleen,” indicating that the primary pathological mechanism of obesity involves the impaired transportation and transformation functions of the spleen. Dysfunction of the spleen results in the disruption of the small intestine’s ability to separate the pure from the impure and the large intestine’s function of transmission. Phlegm-dampness is considered a yin pathogenic factor, which tends to impair yang functions. According to The Pulse Causes Treatment (Mai Yin Zheng Zhi), “When the spleen fails in its transportation duties, although food enters the stomach, it cannot be properly digested and transformed into nutrients. The resulting accumulation obstructs pathways, causing impurities to mix with purified substances. Dampness accumulates into heat, and heat generates further dampness, leading to a depletion of yin fluids over time. This imbalance of cold and heat, deficiency and excess, forms a complex and refractory condition.”

Given this intricate pathology, TCM treatment for obesity should balance cold and heat, combining elimination and supplementation strategies. WMW exemplifies this principle by harmonizing pungent, sour, bitter, and sweet flavors. It employs the sour property of Wu Mei (Mume Fructus) in combination with warming herbs such as Fu Zi (Aconiti Lateralis Radix Preparata), Gan Jiang (Zingiberis Rhizoma), Gui Zhi (Cinnamomi Ramulus), Xi Xin (Asari Radix et Rhizoma), and Shu Jiao (Zanthoxyli Pericarpium) to disperse cold through warmth and regulate the interior. To counteract excessive pungency, bitter herbs like Huang Lian (Coptidis Rhizoma) and Huang Bai (Phellodendri Chinensis Cortex) are added to clear heat and expel dampness. Additionally, Ren Shen (Ginseng Radix) and Dang Gui (Angelicae Sinensis Radix) are included to strengthen vital energy and replenish deficiencies. Thus, WMW achieves a dynamic balance of cold and heat, elimination and supplementation, making it particularly suited to address the chronic and complex pathology of obesity, characterized by the coexistence of cold and heat, deficiency, and excess.

Our previous studies demonstrated that high doses of WMW can reduce the body weight of obese mice in a short period by decreasing the mass of white adipose tissue, while also enhancing the function of brown adipose tissue in mice receiving WMW intervention [25]. We observed that WMW reduces the body weight of obese mice, consistent with our previous findings. The food intake in both the high-dose WMW and low-dose WMW groups was lower than that in the HFD + Vehicle group, suggesting that appetite suppression might be one of the mechanisms through which WMW exerts its anti-obesity effects. However, the reduction in body weight in the low-dose WMW group was significantly less than the decrease in food intake during the treatment period. Additionally, while the food intake was similar between the high-dose and low-dose WMW groups, the body weight of mice in the low-dose WMW group was notably lower than that of the high-dose group. These findings indicate that WMW, particularly at a low dose, may exert anti-obesity effects through mechanisms beyond appetite suppression. Additionally, we noticed that the food intake of the model group was lower than that of the control group, likely due to the higher energy density of the high-fat diet causing a quicker feeling of satiety compared to regular feed.

To comprehensively assess the effects of WMW on the quantity and activity of brown adipose tissue, we weighed, performed transmission electron microscopy scans, and conducted PET-CT scans on the scapular brown adipose tissue, followed by statistical analyses of related parameters of its mitochondria and lipid droplets. The scapular region is one of the most abundant areas of brown adipose tissue in mammals. Brown adipose cells in this area are dense and active, making it suitable for various experimental analyses. Studies have shown that the response of scapular brown adipose tissue to cold stimulation and other metabolic demands is similar to that of brown adipose tissue throughout the body, thus reflecting the overall metabolic state [50]. Therefore, we chose scapular brown adipose tissue for our brown adipose tissue-related research. The results showed that WMW significantly increased the weight of scapular brown adipose tissue and reduced its lipid droplet content. This indicates that WMW can increase the quantity of brown adipose tissue and reduce its fatty acid content. Immunohistochemical results showed that WMW significantly increased the expression levels of PGC1α and UCP1. PGC1α is a transcription factor that promotes mitochondrial biogenesis and enhances function, thereby increasing the metabolic activity of brown adipose cells; UCP1 mediates heat loss during fatty acid oxidation, promoting heat production in the body. The increase in these proteins indicates that WMW has biological activity in promoting the metabolism and thermogenesis of brown adipose tissue.

Some studies suggest that although brown adipose tissue has potential in energy consumption, its role in overall energy balance and weight control may be relatively limited because the amount of brown adipose tissue in the human body is relatively small. Even with high activity, it may not be sufficient to produce a significant weight loss effect [51]. Considering that white adipose tissue occupies a higher proportion in the body, promoting the browning of white adipose tissue may be a more convincing mechanism for WMW in treating obesity. The browning of white adipose tissue is a physiological process where white adipose cells transform into cells with characteristics of brown adipose cells through a series of molecular and cellular mechanisms. Specifically, after browning, white adipose cells exhibit metabolic activity similar to that of brown adipose cells, capable of rapidly consuming energy and generating heat [52]. Existing research has proven that by inducing the browning of white fat, obesity can be effectively treated [53]. However, compared to the increase in the number of brown adipose tissue in the scapular region, the browning of white adipose tissue is difficult to observe under a microscope. Therefore, we detected the expression of brown adipose markers in white adipose tissue, including UCP1, PGC-1α, CIDEA, and Elovl3. Our experimental results indicate that both high and low doses of WMW feeding can reverse the reduction in UCP1, PGC-1α, CIDEA, and Elovl3 expression in the white adipose tissue of HFD-fed mice, suggesting that WMW can increase the browning of white adipose tissue in obese mice, thereby achieving the effect of treating obesity.

HSF1 is a major member of the heat shock factor family and is activated under stress conditions (such as high temperature, oxidative stress, etc.) and regulates the expression of a series of heat shock proteins to maintain cell homeostasis [54]. Recent research has shown that HSF1 plays an important role in the differentiation and metabolic activity of brown fat [55]. Specifically, firstly HSF1 is activated by dissociating and polymerizing with Heat shock protein 90 (HSP90), HSP70 and HSP40 and protein modification, and then binds to PGC1α, thereby activating the thermogenic program of BAT [56]. Secondly, HSF1 can increase the transcription of Hnrnpa2b1 (A2b1), thereby increasing the mRNA stability of key metabolic genes such as UCP1 and promoting the browning of white fat [45]. In summary, HSF1 promotes the thermogenic capacity of brown adipose tissue by regulating the activity of heat shock proteins and PGC1α, while also increasing the stability of key metabolic genes, thus driving the conversion of white adipose tissue to brown adipose tissue. These functions suggest that HSF1 has significant potential in regulating adipose tissue metabolism and addressing obesity. In our experiments, we found that the expression of HSF1, heat shock proteins, and their downstream target A2B1 were significantly reduced in obese mice compared to control groups, which is consistent with other studies. Based on this, we observed that WMW can increase the expression of HSF1 in obese mice. We then investigated whether HSF1 is a key target for WMW's effects on brown adipose tissue by simultaneously administering WMW and an HSF1 inhibitor. DTHIB, which promotes HSF1 degradation by specifically binding to HSF1, was chosen as the HSF1 inhibitor [57]. Our experimental results showed that intraperitoneal injection of DTHIB effectively inhibited the expression of HSF1. Subsequently, we found that inhibiting HSF1 expression significantly weakened the effects of WMW on increasing brown adipose tissue activity and quantity as well as the browning of white adipose tissue, indicating that HSF1 is a key target for WMW in these processes.

Immunohistochemistry and fluorescence data showed that WMW increases the expression of HSPs and A2B1 along with HSF1, and this effect was abolished when the HSF1 inhibitor was introduced. Literature shows that activation of HSF1 can significantly increase the expression of HSPs [58]. Another study indicates that HSF1 responds to pathological proteins in neurodegenerative diseases by increasing heat shock protein expression [59]. Additionally, HSF1 directly induces A2B1 expression by binding to the promoter region of the A2B1 gene, thereby increasing its transcription level, suggesting that HSF1 directly regulates the expression of HSPs and A2B1 [60]. The effect of WMW on increasing the expression of HSPs and A2B1 is thus associated with HSF1.

To identify which chemical constituents in WMW interact with HSF1 to exert their effects, we performed molecular docking between the individual chemical components of WMW identified in previous studies and HSF1. We found that 11 chemical constituents showed strong affinity for HSF1. Considering the mechanism of action of HSF1, we have reason to believe that the various chemical components of WMW may play different roles in different physiological processes. These roles may include enhancing the stability of HSF1, promoting interactions between HSF1 and various target proteins, and facilitating the mobility of HSF1. Moreover, several studies indicate that butyrate, one of the SCFAs, can increase HSF1 expression. Furthermore, we also found that WMW can reverse the reduction of acetate and isobutyrate levels in the intestines and the serum of obese mice. Acetate is one of the main SCFAs produced by the fermentation of dietary fiber by gut microbiota, which can regulate intestinal pH, inhibit the growth of harmful bacteria, and promote the proliferation of beneficial bacteria [61]. Additionally, acetate has positive effects on host metabolic health by providing energy and regulating lipid metabolism [62]. Although isobutyrate is present in lower amounts in the gut, studies have shown that it works together with other SCFAs to regulate gut microbiota balance, contributing to the maintenance of intestinal health [63, 64]. Therefore, WMW’s ability to promote the production of beneficial SCFAs suggests other potential positive effects on host metabolic health that warrant further investigation. In summary, through DIA proteomics screening, we discovered the primary mechanisms by which WMW regulates brown adipose tissue and confirmed that HSF1 is its main target. Further in vivo inhibitor experiments, molecular docking and SCFAs analysis validated the critical role of HSF1 in the regulation of brown adipose tissue by WMW.

In this study, we used the same WMW dosage as in our previous research. Based on the human-animal dosage conversion formula, the clinical equivalent dose of WMW for mice was calculated to be 0.96 g/ml. Given that our previous experiments found that 1.92 g/ml WMW had a good weight-reducing effect, we set 1.92 g/ml as the high-dose group. However, our experiments showed that LWMW had a better therapeutic effect than HWMW. First, OGTT experiments showed that LWMW significantly improved the overall glucose tolerance of high-fat diet mice, whereas HWMW did not, a phenomenon not observed in previous experiments. Further statistical data indicated that both HWMW and LWMW could reduce fasting blood glucose levels in obese mice, but only LWMW significantly improved postprandial blood glucose control. This suggests that while WMW can improve basic insulin function, HWMW might impair the body's cellular response to blood glucose or insulin signaling pathways.

Secondly, we observed that LWMW significantly increased the thermogenesis of scapular brown adipose tissue in mice and protected mitochondrial function. However, the brown adipose tissue thermogenesis and mitochondrial integrity in the HWMW group did not significantly increase, and the expression of HSF1, HSPs, and A2B1 in both brown and white adipose tissues was lower than in the LWMW group. This indicates that the HWMW group experienced impaired brown adipose tissue thermogenesis and reduced browning of white adipose tissue. The possible reason might be that excessively high doses of WMW damage mitochondrial morphology or inhibit fat thermogenesis pathways, thereby reducing the function of brown adipose tissue. These adverse effects could partially offset the increase in HSF1 expression, subsequently reducing the expression of HSPs and A2B1.

In summary, although previous experiments confirmed that high doses of WMW can reduce body weight in obese mice in the short term by decreasing the mass of white adipose tissue, considering its potential adverse effects on postprandial blood glucose control and brown adipose tissue thermogenesis, we believe that LWMW is a more preferable strategy for long-term weight management. However, our experiments also revealed that both HWMW and LWMW reduced lipid droplet storage in BAT, as evidenced by improvements in the lipid droplet ratio, number, and average area in BAT at both concentrations. We speculate that WMW, regardless of concentration, can reduce lipid droplet storage in adipose tissues, including both white and brown adipose tissue. Since WAT primarily consists of lipid droplets, HWMW may reduce the mass of WAT by decreasing the size and number of lipid droplets, consistent with the findings from our previous experiments. Although HWMW did not protect mitochondrial function, the reduction in lipid droplets led to an increase in the proportion of mitochondrial area. Given the risk of HWMW impairing brown adipose tissue thermogenesis, the second part of our animal experiments involved co-administering LWMW with an HSF1 inhibitor.

Our study has some limitations, including not exploring whether WMW promotes HSF1 interaction or transport with target proteins and the lack of a comparison between the efficacy of individual TCM components and WMW. Future studies should delve deeper into these aspects. In conclusion, WMW primarily increases the activity and quantity of brown adipose tissue and promotes the browning of white adipose tissue through HSF1, but LWMW better avoids the impairment of brown adipose tissue thermogenesis, making it more clinically promising for maintaining weight after weight loss.

## Supplementary Information

Supplementary Material 1

## Data Availability

The datasets generated and analyzed during the current study are available from the corresponding authors on reasonable request.

## Abbreviations

AUC: Area under the curve
BAT: Brown adipose tissue
CIDEA: Cell death-inducing DNA fragmentation factor-alpha-like effector A
DDA: Data dependent acquisitioned
DEPs: Differentially expressed proteins
DÍA: Data independent acquisition
Elovl3: ELOVL fatty acid elongase 3
FDG: Fluorodeoxyglucose
GIP: Glucose-dependent insulinotropic polypeptide
GLP-1: Glucagon-like peptide-1
HFD: High-fat diet
HSF1: Heat shock factor
HSP40: Heat shock protein 40
HSP70: Heat shock protein 70
HSP90: Heat shock protein 90
HWMW: High-dose Wu-Mei-Wan
Hnrnpa2b1: Heterogeneous nuclear ribonucleoprotein A2/B1
ITT: Insulin tolerance test
LC–MS/MS: Liquid chromatography-tandem mass spectrometry
LWMW: Low-dose Wu-Mei-Wan
ND: Normal diet
OGTT: Oral glucose tolerance test
PCA: Principal component analysis
PET: Positron emission tomography
PGC-1α: Peroxisome proliferator-activated receptor gamma coactivator 1-alpha
RT-qPCR: Real-time quantitative polymerase chain reaction
SCFAs: Short chain fatty acids
SGLT-2: Sodium–glucose co-transporter-2
SPF: Specific pathogen free
UCP1: Uncoupling protein 1
UPLC-ESI–MS/MS: Ultra-performance liquid chromatography-electrospray ionization-tandem mass spectrometry
WAT: White adipose tissue
WMW: Wu-Mei-Wan
