# Supplementary figures and images for: Wu-Mei-Wan enhances brown adipose tissue function and white adipose browning in obese mice via upregulation of HSF1
Source: Chin Med. 2025 Jan 3;20:1. doi: 10.1186/s13020-024-01053-2 (PMC11697821; doi:10.1186/s13020-024-01053-2)

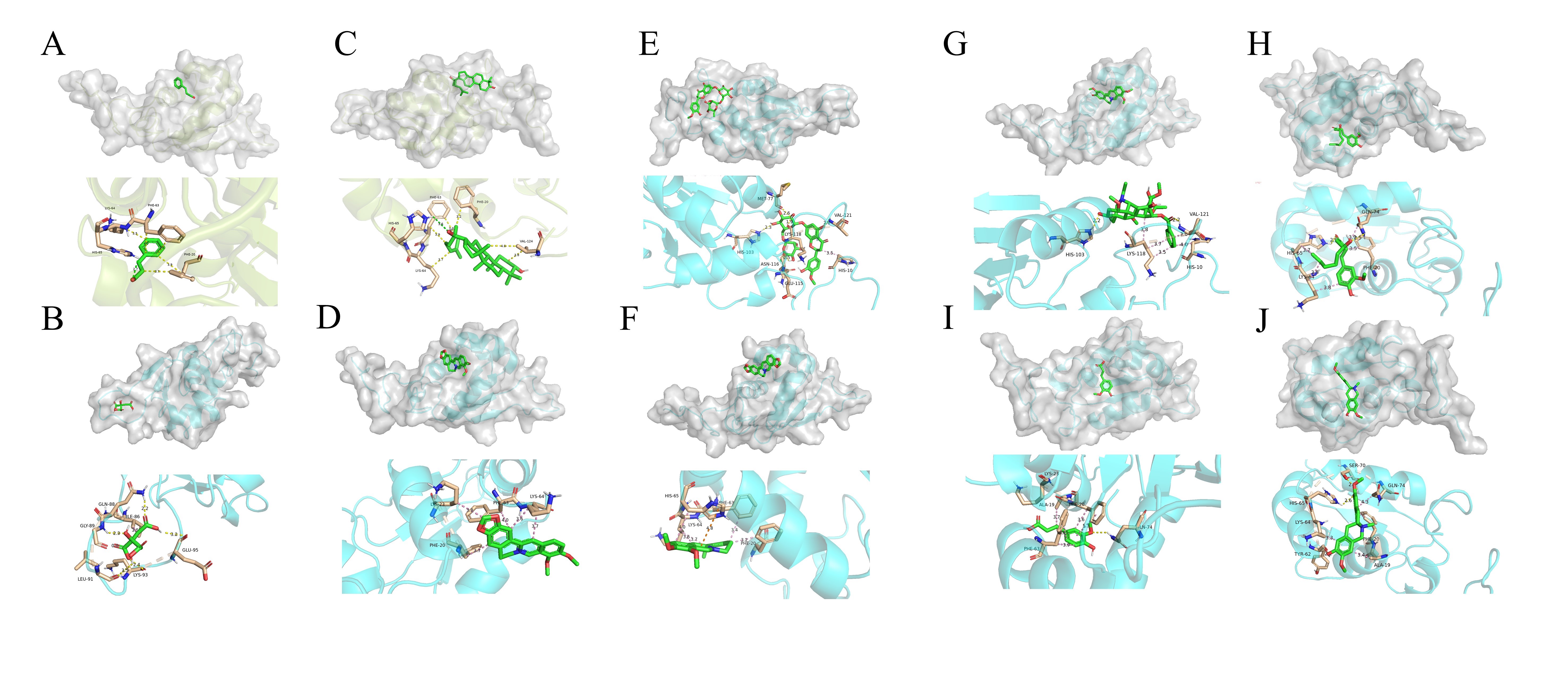

Supplement: Supplementary file 1 — Supplementary Material 1 [file 13020_2024_1053_MOESM1_ESM.jpg]
